# Management of complete intra-articular distal femur and patellar fractures in an achondroplastic young adult; small is challenging’ revisited: a case-report

**DOI:** 10.1186/s13256-024-04566-4

**Published:** 2024-05-11

**Authors:** Hussein Samir Elrukby, Khalid Mohamed Abd Elhafiz Mohamed, Elamin Mohamed Elamin Hamed

**Affiliations:** 1 Department of Surgery, Faculty of Medicine, University of Gezira, Wad-Madani, Sudan; 2 Department of Orthopedic Surgery, Faculty of Medicine, Alzaiem Alazhari University, Khartoum, Sudan; 3 Gezira Center For Orthopedic Surgery and Traumatology, Wad Madani, Sudan

**Keywords:** Achondroplasia, Distal femur, Patella, Fracture

## Abstract

**Background:**

People with achondroplasia exhibit distinct physical characteristics, but their cognitive abilities remain within the normal range. The challenges encountered during surgical procedures and perioperative care for achondroplastic individuals, are underrepresented in the existing literature.

**Case presentation:**

In this report, the management of a 26-year-old North-African achondroplastic male is highlighted. The patient suffered a complete intra-articular distal femur fracture (AO/OTA 33-C1) and an ipsilateral patella fracture (AO/OTA 34-C1). The patient’s unusual anatomical variations and the lack of suitable orthopedic implants posed significant surgical challenges, particularly in the context of a resource-limited developing country. Facial and spinal deformities, which are common in patients with achondroplasia, further complicated the anesthetic approach.

**Conclusions:**

The limited information on operative management of fractures in achondroplastic patients necessitated independent decision-making and diverging from the convenient approach where clear guidance is available in the literature.

## Background

In clinical practice, Commonplace fractures occasionally manifest in exceptional scenarios. We encountered a situation involving a young achondroplastic adult with intra-articular fractures

Achondroplasia is the most common cause of disproportionate skeletal dysplasia. It leads to abnormally short stature, commonly referred to as dwarfism [1]. The genetic mutation—that inhibits subchondral bone growth—involves the gene encoding fibroblast growth factor receptor 3 (FGFR3) [2]. Defective endochondral bone formation results in a wide range of skeletal abnormalities. They include rhizomelic short-limbed stature, flared metaphyses, metaphyseal angulation at the knee joint, and genu-vara [2].

Sleep apnea, neural foraminal compressions, spinal deformities, and the increased risk of cardiopulmonary morbidities in achondroplastic patients contribute to the significant anesthetic challenges faced during surgical procedures [3].

The clinical diagnosis of achondroplasia is typically straightforward, with few differential diagnoses [3].

Individuals with achondroplasia have normal intelligence, and most of them can expect a normal lifespan. However, access to appropriate healthcare services is crucial for minimizing complications [1].

A multidisciplinary team approach, along with active family participation in decision-making regarding achondroplasia health-related issues, is recommended [1].

## Clinical presentation

A young adult North African male was referred to our trauma center following a road traffic accident. The patient’s height measured 125 cm, and he weighed 44 kg. He displayed typical morphological features consistent with achondroplasia, including frontal bossing, megalocephaly, a depressed nasal bridge, mandibular enlargement, rhizomelic limb shortening, and thoracolumbar kyphoscoliosis [3].

The patient was not receiving formal follow-up care for health issues related to achondroplasia due to the unavailability of such services in his locality. Additionally, both the patient and the family accept the patient’s unique appearance, having encountered similar cases within their own family.

The patient’s neurovascular exam revealed normal findings, except for the left knee which was painful and swollen.

The anesthetic assessment revealed minimal cervical range of motion restriction, adequate mouth opening (Mallampati score 3), and thoracolumbar kyphoscoliosis.

## Investigations

**Fig. 1:**
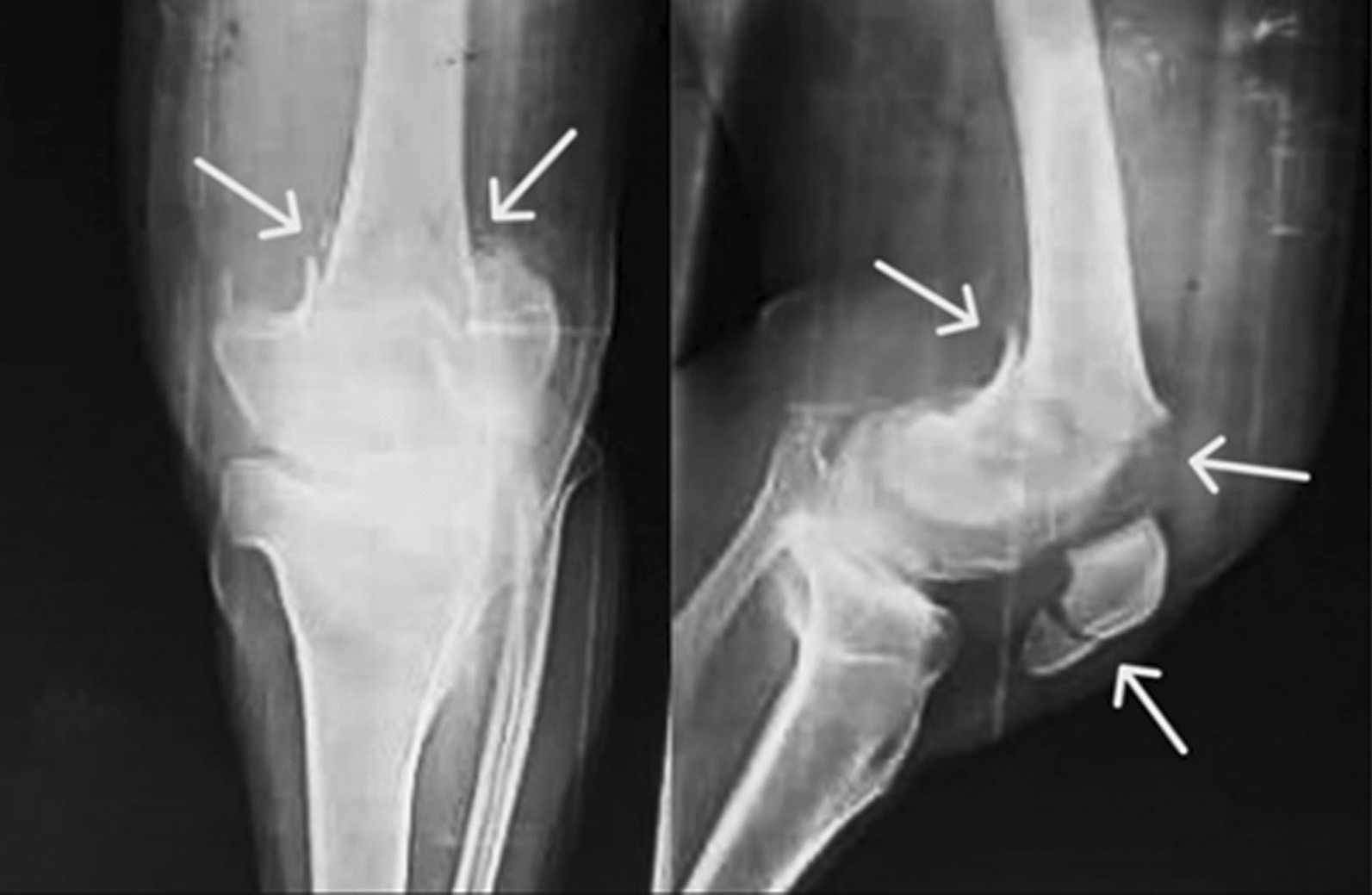
X-ray images showing the distal femoral and patellar fractures as denoted by the arrow indicators

Knee X-rays revealed complete intra-articular fractures affecting both condyles of the left femur (classified as AO/OTA 33-C1). Additionally, there was a concurrent ipsilateral patellar fracture (classified as AO/OTA 34-C1) (Fig. 1).

**Fig. 2:**
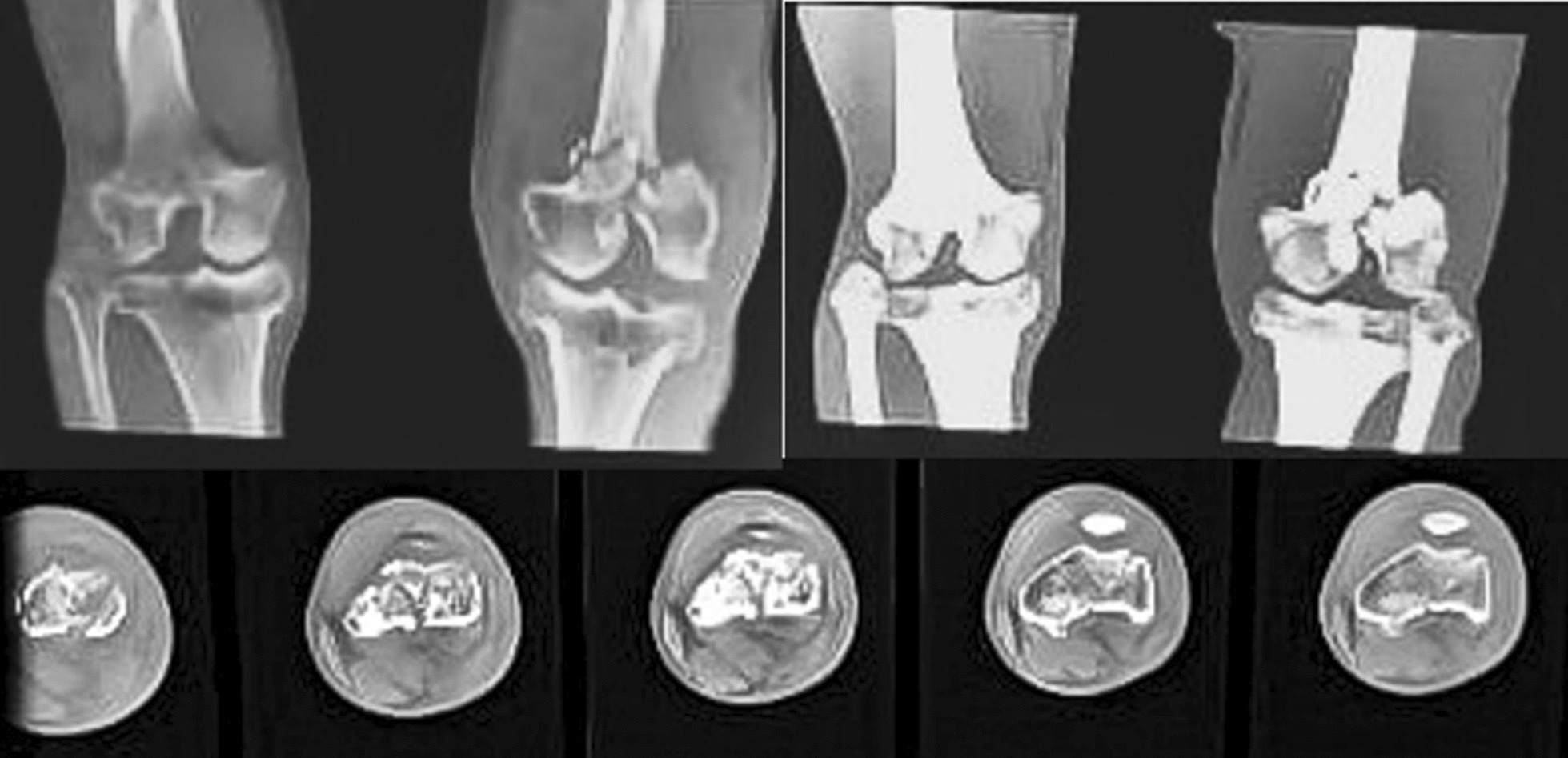
Computed tomography of the left knee showing the fractured distal femoral condyles

A CT scan with 3D reconstruction showed minimal comminution of the fracture (Fig. 2).

Blood parameters, including complete blood counts, serum creatinine levels, serum calcium levels, and serum phosphate levels, were normal. The patient’s cardiovascular and respiratory evaluations also yielded normal results, including a normal echocardiogram.

## Treatment

In the case of a patient with rhizomelic thighs, none of the available tourniquet cuffs were applicable without compromising the surgical field. Consequently, we decided not to utilize them. Instead, we maintained meticulous hemostasis throughout the surgery.

The surgery was performed under spinal anesthesia, the patient was supine, and the left knee was flexed to 30 degrees. An anterior midline incision and a lateral parapatellar approach were employed.

**Fig. 3:**
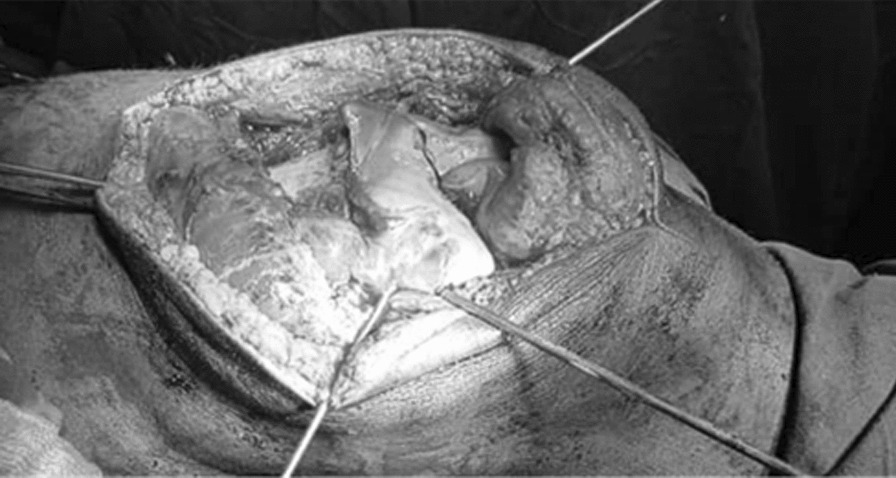
Medical photo showing the preliminary fixation of the condyles

After careful assessment of the atypical anatomy of the fractured distal femoral condyles, successful reduction of the parts was achieved. Subsequently, preliminary fracture fixation using three crossing K-wires was performed (Fig. 3).

Both the lateral and medial menisci and the anterior cruciate ligament were intact.

Intraoperative templating and swift discussion regarding the available implants led us to choose a precontoured medial tibial plateau L-plate. This plate was further contoured intraoperatively to match the anatomy of the lateral aspect of the distal femur.

**Fig. 4:**
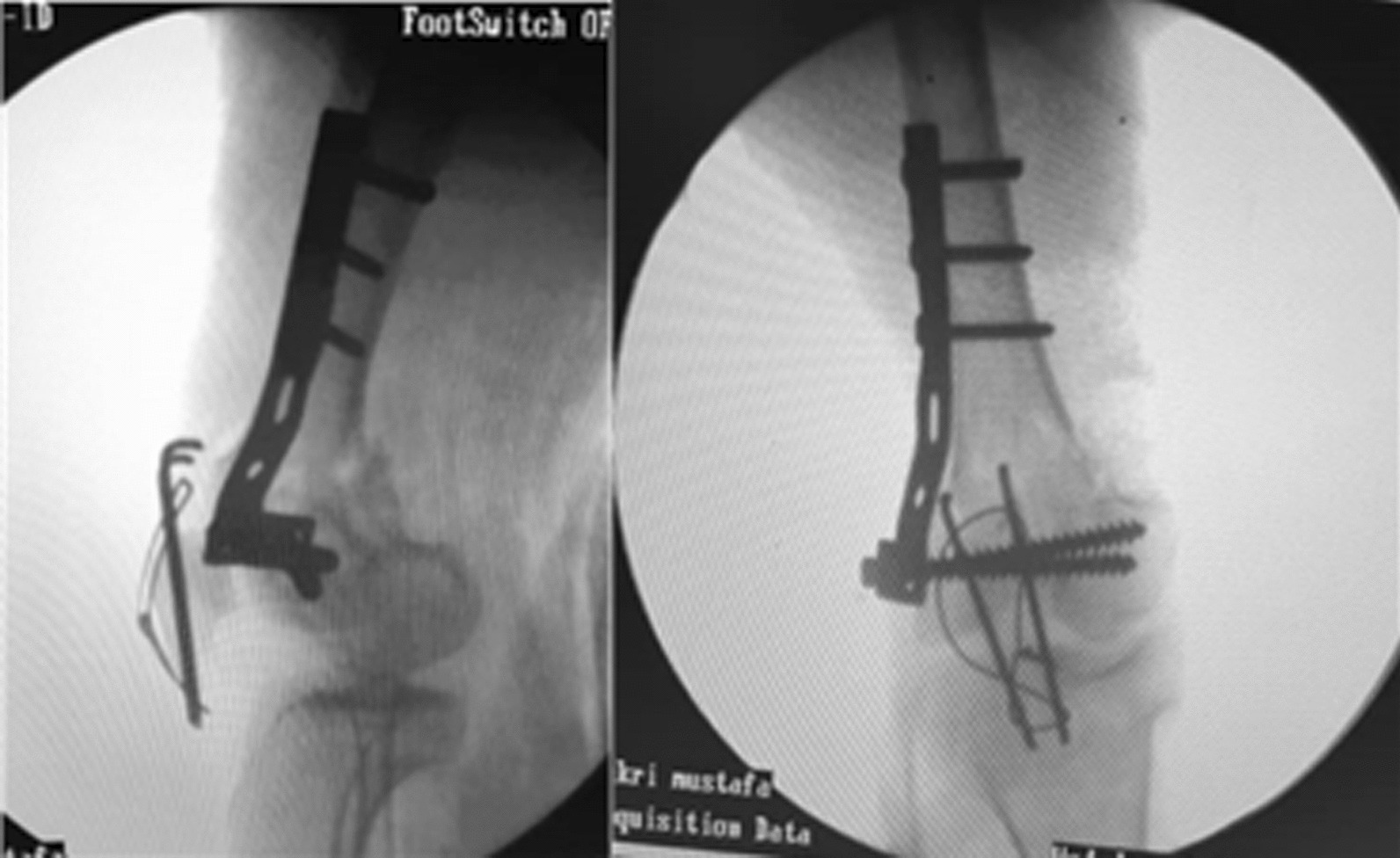
Intraoperative C-arm images showing the final fracture fixation

With C-arm assistance, the stability of the construct was confirmed. The patellar fracture was fixed by cerclage compression wiring, and normal patellar tracking was confirmed (Fig. 4).

The decision was made against the insertion of a drain.

A posterior slab was applied for two weeks to facilitate adequate wound and soft tissue healing.

## Outcomes

The wound healed without complications. Partial weight-bearing was initiated at 6 weeks, with advice to gradually increase weight under the supervision of a physiotherapist. Due to financial constraints, the patient could not stay in the city for an extended period and returned to his locality 12 weeks after the surgery. The patient was provided telephone numbers to contact the surgeon and the physiotherapist. In-person follow-up was scheduled at 6 months and 1 year after the surgery.

The patient was discharged from follow-up at one year. Both the right and left knees exhibited similar ranges of motion. The construct was stable, and the fractures exhibited satisfactory evidence of healing. At discharge, the patient was encouraged to maintain unrestricted mobility.

**Fig. 5:**
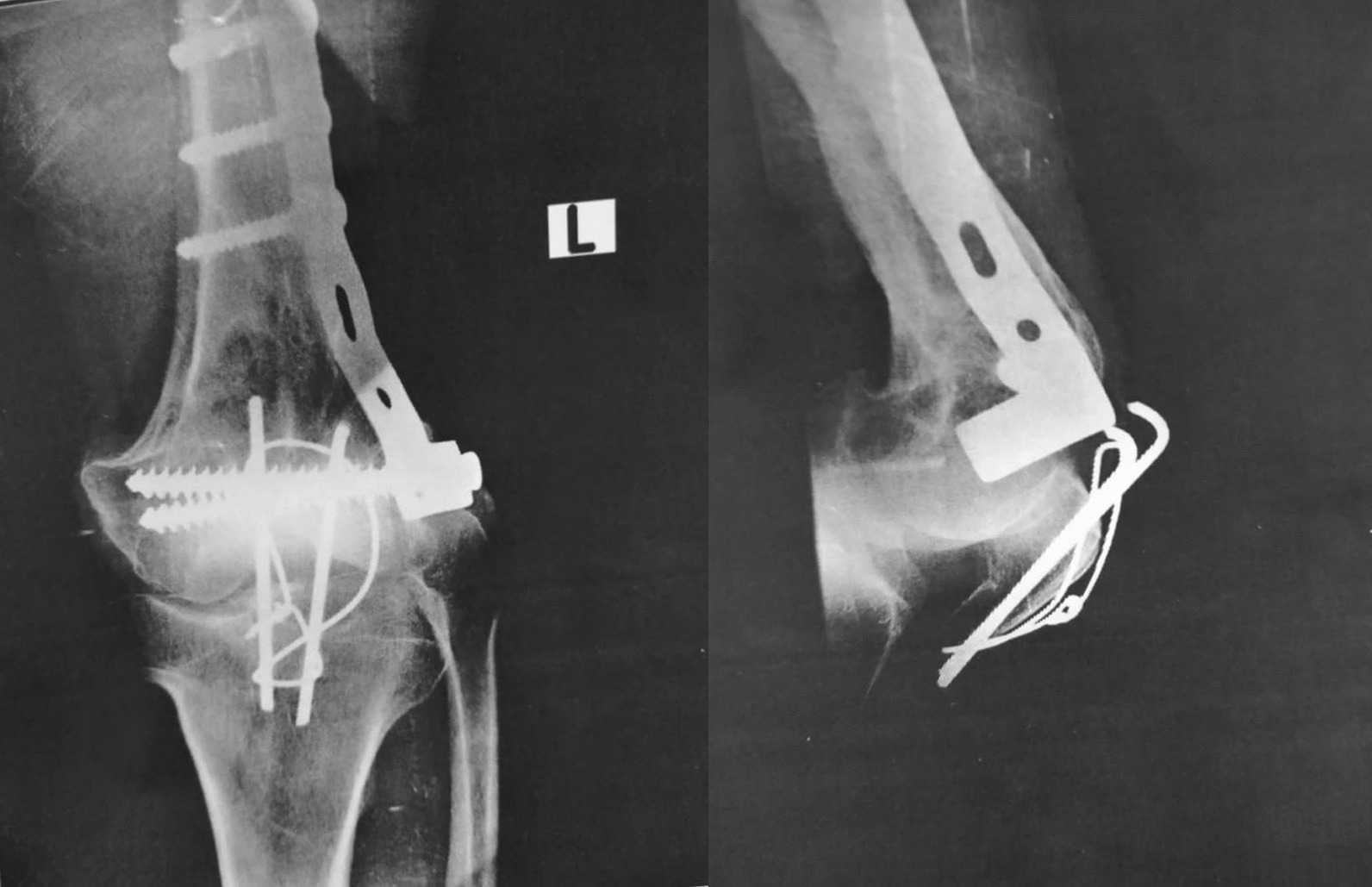
3 years post operative X-ray images showing the consolidation of the fractures

Three years post-surgery, the patient visited the center and expressed no complaints. During this visit, we took the opportunity to obtain X-rays (Fig. 5).

The patient expressed satisfaction with the outcomes of his fracture treatment and reported no decline in previous activities when asked about the impact on the quality of his life.

## Discussion

In a developing country where resources significantly influence decision-making, we rely heavily on the variety of interventions described in the literature to select the most suitable procedures for our context. This approach allows us to optimize patient care while navigating resource constraints.

The management of fractures following trauma in achondroplastic patients is underrepresented in the literature.

We encountered a complex intra-articular fracture of the distal femur (AO/OTA 33-C1), along with a concurrent ipsilateral patellar fracture (AO/OTA 34-C1). We heavily relied on CT scanning with 3D reconstruction to understand the fracture geometry. Addressing this fracture pattern necessitates anatomical reduction, rigid fixation, and early mobilization [4, 5].

Successful spinal anesthesia with low doses of anesthetic drugs has been described in several case reports on achondroplastic patients undergoing surgical interventions [6, 7].

The literature outlines several implant options for treating distal femoral fractures [8]. In light of the patient’s atypical bony morphology and the unavailability of patient-specific implants, we prepared a range of fixation devices for the upcoming surgery. Our preparations included plates, dynamic compression screws (DCSs), wires, and Ilizarov frames. The latter served as our final option, ensuring that we were well- equipped to address any challenges during the procedure.

We concur with Murphy *et al*. that the shortened thighs (rhizomelia) of achondroplastic patients preclude the convenient use of tourniquets [2]. Instead, it’s crucial to maintain meticulous hemostasis to proactively manage any concerns related to excessive blood loss.

The distinctive morphology of the femoral condyles requires careful reduction of the fractured segments. The preliminary fixation of the condylar fractures served as a foundation for intraoperative implant templating, facilitating decisions regarding implant selection, and fitting to the distal femoral topography. None of the prepared implants were an optimal fit for the bony surface. Finally, we decided to utilize a precontoured medial tibial plate, which was further intraoperatively contoured to achieve precise fixation of the intercondylar fractures.

The literature includes two case reports that closely resemble the currently described situation. However, our case stands out due to the complete intraarticular nature of the fracture in a young, active adult. This necessitated precise anatomical reduction with absolute rigidity of fixation, which diverges from the treatment approaches described in the two existing case reports. Specifically, Murphy *et al*. employed three percutaneous screws to address a partially articular distal femur (AO/OTA 33B2) fracture in an elderly achondroplastic individual with significant comorbidities [2]. Another report addressed a supracondylar extraarticular distal femur (AO/OTA 33A3) fracture and employed a humeral nail for fixation. However, this approach does not align with our patient’s fracture geometry [9]. Given these distinctions, our approach prioritized anatomical reduction and rigid fixation to optimize outcomes for this young and active patient. Our approach underscores the importance of tailoring treatment to individual circumstances, even when the literature provides limited guidance. We opted for an open surgical approach to address both the distal femoral and patellar fractures, ensuring accurate anatomical reduction. The anterior longitudinal midline incision and lateral parapatellar approach combined with the patellar fracture provided excellent exposure of both the medial and lateral sides of the distal femur. Furthermore, it permits the possibility of subsequent revision to total knee arthroplasty (TKA), using the same approach if necessary [10, 11].

No drain was placed following meticulous hemostasis, in line with recommendations from the literature advocating against its use [12].

While individuals with achondroplasia typically exhibit normal intelligence, we recognize that their physical differences can already pose significant challenges. Our approach aimed to minimize any additional difficulties they may experience by carefully considering the most effective management options.

The patients’ commitment to pre- and postoperative instructions was outstanding. Patient compliance likely plays a crucial role in achieving positive outcomes.

## Conclusion

- Encountering rare cases: We mitigated uncertainties by consulting the available literature and engaging in discussions with the surgical team.
- Applying principles of fracture fixation: Despite the unusual situation, we carefully considered providing the best possible choice according to the principles of fracture management.
- Recognizing anesthetic risks: We involved the anesthesiologist early in the process, given the special risks associated with achondroplasia (such as spine deformities, difficult intubation, and cardiothoracic complications).
- Planning and templating: In achondroplasia patients, standard implants may not fit their unique skeletal morphology. Therefore, intraoperative fitting and contouring of implants are essential for optimal fixation.
- Healing of fractures: Fractures of the distal femur in patients with achondroplasia tend to heal well [2, 9].

## Data Availability

Data sharing is not applicable to this article as no datasets were generated or analyzed during the current study.

## Abbreviation

AO/OTA: Arbeitsgemeinschaft für Osteosynthesefragen/Orthopedic Trauma Association
